# Hypoxia Exacerbates Inflammatory Acute Lung Injury *via* the Toll-Like Receptor 4 Signaling Pathway

**DOI:** 10.3389/fimmu.2018.01667

**Published:** 2018-07-23

**Authors:** Gang Wu, Gang Xu, De-Wei Chen, Wen-Xiang Gao, Jian-Qiong Xiong, Hai-Ying Shen, Yu-Qi Gao

**Affiliations:** ^1^College of High Altitude Military Medicine, Institute of Medicine and Hygienic Equipment for High Altitude Region, Army Medical University, Chongqing, China; ^2^Key Laboratory of High Altitude Medicine, People’s Liberation Army, Chongqing, China; ^3^Department of Pathophysiology, College of High Altitude Military Medicine, Army Medical University, Chongqing, China; ^4^Intensive Care Unit, Southwest Hospital, Army Medical University, Chongqing, China; ^5^Robert Stone Dow Laboratories, Legacy Research Institute, Legacy Health, Portland, OR, United States

**Keywords:** acute lung injury, hypoxia, gene chip, hypoxia-inducible factor-1α, lipopolysaccharide, toll-like receptor-4

## Abstract

Acute lung injury (ALI) is characterized by non-cardiogenic diffuse alveolar damage and often leads to a lethal consequence, particularly when hypoxia coexists. The treatment of ALI remains a challenge: pulmonary inflammation and hypoxia both contribute to its onset and progression and no effective prevention approach is available. Here, we aimed to investigate the underlying mechanism of hypoxia interaction with inflammation in ALI and to evaluate hypoxia-inducible factor 1 alpha (HIF-1α)—the crucial modulator in hypoxia—as a potential therapeutic target against ALI. First, we developed a novel ALI rat model induced by a combined low-dose of lipopolysaccharides (LPS) with acute hypoxia. Second, we used gene microarray analysis to evaluate the inflammatory profiles of bronchi alveolar lavage fluid cells of ALI rats. Third, we employed an alveolar macrophage cell line, NR8383 as an *in vitro* system together with a toll-like receptor 4 (TLR4) antagonist TAK-242, to verify our *in vivo* findings from ALI animals. Finally, we tested the therapeutic effects of HIF-1α augmentation against inflammation and hypoxia in ALI. We demonstrated that (i) LPS upregulated inflammatory genes, tumor necrosis factor alpha (TNF-α), interleukin-1 beta (IL-1β), and interleukin-6 (IL-6), in the alveolar macrophages of ALI rats, which were further enhanced when ALI combined with hypoxia; (ii) hypoxia exposure could further enhance the upregulation of alveolar macrophageal TLR4 that was noticed in LPS-induced inflammatory ALI, conversely, TLR4 antagonist TAK-242 could suppress the macrophageal expression of TLR4 and inflammatory cytokines, including TNF-α, IL-1β, and IL-6, suggesting that the TLR4 signaling pathway as a central link between inflammation and hypoxia in ALI; (iii) manipulation of HIF-1α *in vitro* could suppress TLR4 expression induced by combined LPS and hypoxia, *via* suppressing promoter activity of the TLR4 gene; (iv) preconditioning augmentation of HIF-1α *in vivo* by HIF hydroxylase inhibitor, DMOG excreted protection against inflammatory, and hypoxic processes in ALI. Together, we see that hypoxia can exacerbate inflammation in ALI *via* the activation of the TLR4 signaling pathway in alveolar macrophages and predispose impairment of the alveolar-capillary barrier in the development of ALI. Targeting HIF-1α can suppress TLR4 expression and macrophageal inflammation, suggesting the potential therapeutic and preventative value of HIF-1α/TLR4 crosstalk pathway in ALI.

## Introduction

Acute lung injury (ALI) refers to a clinical syndrome characterized by bilateral lung injury, severe diffuse failure of the lung, or hypoxemia caused by non-cardiogenic pulmonary edema (1). ALI is the most severe form of diffuse lung disease and has a high (40–60%) morbidity and mortality rate (2). Sepsis is the primary etiology of ALI (3) and a common admission to the intensive care unit (4); it induces pulmonary inflammation leading to disruption of endothelial–epithelial barriers by surge release of pro-inflammatory cytokines, which in turn increase the permeability of the alveolar-capillary membrane, pulmonary infiltration, and edema (5, 6). Other causes of ALI include trauma (7), aspiration (8), acute pancreatitis (9), drug toxicity (10), etc. Ultimately, gas exchange across the alveolar-capillary membrane becomes severely impaired and acute respiratory failure and hypoxia occur (11). ALI patients may suffer from pulmonary inflammation and hypoxia simultaneously or sequentially (12), as seen in clinical settings. These two pathophysiological processes may interact to mutually contribute to the development of ALI. For example, intestinal bacterial translocation can result in shock lung (13) and generate systemic hypoxia and increasing hypoxic microenvironment by increasing cellular oxygen consumption at the infected site. Also, respiratory infection and inflammation can lead to high altitude pulmonary edema (HAPE) in individuals with acute exposure to hypoxic high altitude (14–16). On the other hand, hypoxia has its own important pathophysiological basis and aftermath, such as in shock (17, 18), heart and/or respiratory failure (19), and high altitude diseases (20), which can affect and interact with the inflammatory responses in ALI. A recent study (21) showed that hypoxia exposure at high altitude significantly increased plasma cytokine levels, tumor necrosis factor alpha (TNF-α), interleukin-1 beta (IL-1β), and interleukin-6 (IL-6), suggesting crosstalk between inflammation and hypoxia.

During sepsis, endotoxin lipopolysaccharides (LPS) activate toll-like receptor 4 (TLR4) signaling pathway and further induce translocation of nuclear factor-kappa B (NF-kappa B) to modulate expression of pro-inflammatory genes, including TNF-α, IL-6, and IL-1β (22). LPS also initiate a pro-inflammatory cascade in immune cells, e.g., neutrophils, monocytes, and endothelial cells (23). By contrast, under acute hypoxic conditions, hypoxia-inducible factor 1 (HIF-1) protects cells against hypoxic stress *via* regulating the expression of more than 100 downstream genes (24), a number of which have been proved to impact the development of inflammation (25). Though HIF-1 function has been extensively studied, its role in the regulation of inflammation is still a debatable point (26–31). In addition, while increasing data suggest possible crosstalk between inflammation and hypoxia (3, 5, 32–34), the underlying mechanisms still remain elusive. This critical gap is of key importance for developing approaches for the prevention and treatment of ALI.

Therefore, we hypothesized that hypoxia can affect LPS-induced inflammatory responses in the development of ALI—if verified—the regulator(s) of this crosstalk may become a therapeutic target for treatment against both hypoxic and inflammatory impairment in ALI. To test our hypothesis, we first developed a novel ALI rat model induced by a combined low-dose of LPS with acute hypoxia. We then evaluated the *in vivo* inflammatory profiles of bronchi alveolar lavage fluid (BALF) cells in ALI rats and *in vitro* changes of alveolar macrophage cell line, NR8383, using gene microarray screening and real-time quantitative PCR (RT-qPCR) analysis. Finally, we tested the therapeutic role of *in vitro* manipulation of TLR4 on NR8383 cells and *in vivo* augmentation of hypoxia-inducible factor 1 alpha (HIF-1α) in our established novel ALI model.

## Materials and Methods

### Materials

The TLR4 antagonist TAK-242, the HIF-1α antagonist PX478, and the HIF prolyl hydroxylase inhibitor DMOG were purchased from the MedChem Express (Shanghai, China). Small interfering RNA (siRNA) sequences for HIF-1α were constructed by GenePharma (Shanghai, China). Dimethyl sulfoxide and LPS (*Escherichia coli* 055: B5) were purchased from Sigma-Aldrich (St. Louis, MO, USA). Rat TNF-α, IL-1β, and IL-6 enzyme-linked immune sorbent assay (ELISA) kits were purchased from RayBiotech (Norcross, GA, USA). The anti-HIF-1α monoclonal antibody was purchased from Abcom (Beverly, MA, USA). The anti-TLR4 monoclonal antibody was purchased from Santa Cruz Biotechnology (CA, USA). The anti-β-actin monoclonal antibody was purchased from Sigma-Aldrich (St. Louis, MO, USA). TLR4 promoter-luciferase was constructed by GenePharma (Shanghai, China). Luciferase activities were assayed 24–48 h after transfection using a luciferase reporter assay system (Promega). Transient transfections were performed with Lipofectamine 3000 (Invitrogen).

### Animals and Treatments

All animal experiments were approved by the intramural Committee on Ethics Conduct of Animal Studies of the Army Medical University in Chongqing, China. Briefly, 8-week-old male Sprague-Dawley rats, weighing 200 ± 20 g, were housed in microisolator cages with specific pathogen-free condition and free access to water and food. The rats were supplied by the Center of Experimental Animal of the Army Medical University in Chongqing, China. Laboratory temperature was 24 ± 1°C, and relative humidity was 40–80%. Before experimentation, rats were housed for 3 days to allow acclimation to the environment. The rats were randomly divided into four experimental groups: (i) CTL group, naïve control rats with normoxia, (ii) LPS group, with LPS treatment with normoxia, (iii) HPO group, with exposure of acute hypobaric hypoxia, and (iv) COMB group, with combined exposures of acute hypobaric hypoxia and LPS, the total number of animals used in each group was 30. For LPS administration, the animals were subjected to a tail vein injection of a 0.5 mg/kg LPS in saline at an injection volume of 0.2 ml (LPS: 1 mg/ml). For exposure to hypobaric hypoxia, the animals were subjected to a decompression chamber for 6 h with an ambient air pressure of 405.35 mmHg (approximately 0.53 atm, or equivalent of 5,000 m altitude, or the equivalent of 11.2% O_2_). The animals in the COMB group that received double-stimulus were first subjected to LPS administration, then the decompression chamber. The rats in the CTL group were subjected to a tail vein injection of 0.2 ml of saline. All animals had no access to food and drinking water during the experiment. Specimen collection was conducted under the same (normal or hypoxic) conditions as the treatment of animal to eliminate artificial modifications. Before specimen collection, rats were subjected to anesthesia with a single dose of chloral hydrate (100 mg/kg, i.p.).

### Measurement of Mean Pulmonary Artery Pressure (mPAP)

The mPAP was measured according to the previously described method (35) after rats were exposed to hypoxia. First, the rats were anesthetized with a single injection of chloral hydrate (100 mg/kg, i.p.). A micro catheter was then inserted through the right external jugular vein into the right ventricle and finally positioned into the pulmonary artery for measurement of mPAP. Blood samples were harvested from rats after mPAP measurement.

### Detection of Arterial Oxygen Saturation of Rats

To investigate the hypoxic status in rats with ALI, arterial blood was sampled from the abdominal aorta for blood gas analysis according to the previously described method (36).

### Evaluation of Bronchoalveolar Lavage Fluid

To determine the inflammatory response in the lungs, BALF was collected for evaluation. Rats were anesthetized with a single dose of chloral hydrate (100 mg/kg, i.p.) and euthanized by lung removal. BALF was obtained from the right lung as follows: first, the left bronchus was ligated with sutures and the right bronchus was cannulated. Then 3 ml saline was introduced into the right lung, which was then gently manipulated, followed by withdrawal of the lavage fluid. This procedure was repeated three times per rat. The BALF was collected by centrifugation and the resultant supernatant was used for biochemical analysis using an automatic microplate reader (Tecan Sunrise, USA). The cell fraction was suspended in PBS and used for (i) the evaluation of inflammatory gene expression profiling and (ii) the total cell counts by hemocytometers.

### Histopathologic Evaluation

Histopathologic evaluation was performed on rats not subjected to BALF collection. Six hours after stimuli exposure, rats were sacrificed and their lungs were collected. After being fixed in 4% buffered formalin for 1 week, the lung tissues were embedded in paraffin and sliced into 5 µm sections. Hematoxylin and eosin (H&E) staining was performed for histopathologic evaluation.

### Assessment of Wet-to-Dry (W/D) Ratio of Lung Tissue

As the W/D ratio of the lung tissue is an important index for assessing the degree of pulmonary edema in ALI (37), we sought to examine the W/D ratio changes after different stimulations. The rats were anesthetized by a single injection of chloral hydrate (100 mg/kg i.p.) and the left lung was removed for determining its wet weight using a precision electronic scale (BSA124S-CW; Sartorius, Germany). Then the lung was placed in an oven and baked at 56°C for 48 h until a constant weight was obtained—as dry weight. The W/D ratio was calculated to represent the water content level of the lung tissue.

### Cell Culture and Treatments

Rat alveolar macrophage-derived cell line NR8383 (Cell Bank of the Chinese Academy of Sciences, Shanghai, China) was cultured in F-12K medium (containing 20% inactivated fetal bovine serum, 50 U/ml penicillin, 50 U g/ml streptomycin) in a humid atmosphere with 5% CO_2_ and 95% air; then cultured cells were randomly divided into four groups for evaluation of gene expression and signaling pathway after exposure to stimuli as follows: (i) CTL group, as naïve control; (ii) LPS group, cells cultured with 10 ng/ml LPS in media under normoxia; (iii) HPO group, cultured with regular media and exposure to 5% O_2_; and (iv) COMB group, cells cultured with the combination of both 10 ng/ml LPS and 5% O_2_ exposure. To abolish the effect of HIF-1α, cells were pretreated with 50 µM PX478 for 20 h under normoxia as described (38). To augment the effect of HIF-1α, cells were pretreated with 1 µM DMOG for 8 h under normoxia as described (39).

### Enzyme-Linked Immune Sorbent Assay

Inflammatory profiles (TNF-α, IL-1β, and IL-6) of BALF, rat plasma, and the culture media of NR8383 cells were evaluated by ELISA. The samples were centrifuged (3,000 *g* for 10 min at 4°C) then stored at −80°C for ELISA testing. The concentrations of TNF-α, IL-1β, and IL-6 were detected by commercially available kit (RayBiotech, GA, USA) per manufacturers’ instructions. Plates were read at 450 nm by an automatic microplate reader (Tecan Sunrise).

### Western Blot Analysis

Whole-cell lysates were obtained by resuspending cell pellets in RIPA buffer (50 mM Tris pH 7.4, 150 mM NaCl, 1% Triton X-100) with freshly added protease inhibitor (Roche). The concentration of protein was measured by a BCA protein assay kit (Pierce, Rockford, IL, USA) according to the manufacturer’s instructions. 50 µg of protein lysate was fractionated by SDS-PAGE and then transferred onto a PVDF membrane. The membrane was blocked with 5% milk at room temperature for 1 h then probed with primary antibodies overnight at 4°C. The membrane was washed with TBST and then incubated with corresponding secondary antibody for 1 h at room temperature. The targeted proteins were visualized with super signal West Pico Chemiluminescent Substrate (Thermo Scientific, USA). The anti-β-actin antibody (Sigma, Cat #A2228) was used as an internal control. Densitometry was performed using Image J (National Institutes of Health, USA). Protein levels were normalized to β-actin.

### Real-Time Quantitative PCR

Total RNA was extracted from BALF cells or cultured NR8383 cells using RNA reagent (Takara Japan), and the first-strand cDNA was synthesized using an MLV reverse transcription kit (Takara, Japan) according to the manufacturer’s instructions. Primers are shown in Table 1. The RT-qPCR reactions were conducted using a Bio-Rad real-time PCR system with the following modified program: initial denaturation at 95°C for 2 min; followed by 40 cycles of denaturation at 95°C for 30 s, annealing at 60°C for 30 s, and amplification at 72°C for 20 s. Products were verified by melting curve analysis and 1.5% agarose gel electrophoresis. The cDNA was used as a template for RT-qPCR using the SYBR green master mix (Applied Biosystems, USA). Gene expression levels were calculated relative to β-actin internal control.

**Table 1:** Oligonucleotide primers of rat used in real-time quantitative PCR.

Gene	Primer sequence (5′–3′) forward	Primer sequence (5′–3′) reverse	Amplicon length (bp)
IL-1β	AGTGAGGAGAATGACCTGTTC	CGAGATGCTGCTGTGAGATT	124
IL-6	GCCAGAGTCATTCAGAGCAATA	GTTGGATGGTCTTGGTCCTTAG	160
TNF-α	ACCATGAGCACGGAAAGCAT	AACTGATGAGAGGGAGCCCA	220
LBP	TGACTACAGTTTGGTGGCGG	TTGGTGTTCAGCCGGATGTT	273
CD14	TCCCACTCTCAGAATCTACC	CACACGCTTTAGAAGGTACT	249
TLR4	CCCTGCCACCATTTACAGTT	TGCCATGCCTTGTCTTCAAT	358
NF-kBia	AGACTCGTTCCTGCACTTGG	TCTCGGAGCTCAGGATCACA	214
β actin	CACCCGCGAGTACAACCTTC	CCCATACCCACCATCACACC	207
CCL3	ATATGGAGCTGACACCCCGA	GGAGGTTTGGGGGTTCCTTG	237

### Identification of Gene Expression Profiling of BALF Cells

For microarray analysis, we pooled the total RNA from three animals in the same treatment group as a pooled RNA sample, and a total of three pooled RNA samples underwent microarray analysis in each group. We used cluster analysis to analyze these 12 pooled RNA samples to find the common genes identified by paired *t*-test. The clustering display was generated by Chip software with two-way data clustering. The gene expression profiles were determined using Gene Chip Rat Genome 230 2.0 Array (Affymetrix). RNA samples were hybridized onto array chips, stained, washed, and scanned according to Affymetrix protocol. The array image and cell intensity files (CEL files) were generated by Affymetrix Gene Chip Command Console; the RNA quality control tests and Gene Chip analysis were conducted by Center for Genomic Services (Hong Kong University). The analysis of data from Gene Chip was conducted using MAS 3.0 Software, Affymetrix Transcriptome Analysis Console Software, and R software (http://www.r-project.org). Filtering was performed to remove background noise with Gene Spring Software. Probes with signals weaker than the 20th percentile of the overall signal were not included in the analysis. Differentially expressed genes were identified by fold change (FC ≥ 2 or FC ≤ 0.5). Over-represented gene ontology (GO) terms and enriched pathways associated with the list of differentially expressed genes were generated by the built-in GO and single experiment analysis of gene spring. Only GO terms and pathways that have more than two entities involved and *p*-value <0.05 were considered.

### Statistical Analysis

The data are presented as mean values with SD of the means. All the cell experiments were repeated three times. Significant differences between two or more groups were analyzed using Student’s *t*-test or one-way analysis of variance by using an SPSS 20.0 software (USA), accordingly. The pictures were drawn by GraphPad Prism software 5.0 (GraphPad, CA, USA) for Windows. A value of *p* <0.05 was considered a significant difference.

## Results

### Novel Rat Model of ALI Induced by LPS Combined With Hypoxia

**Figure 1:**
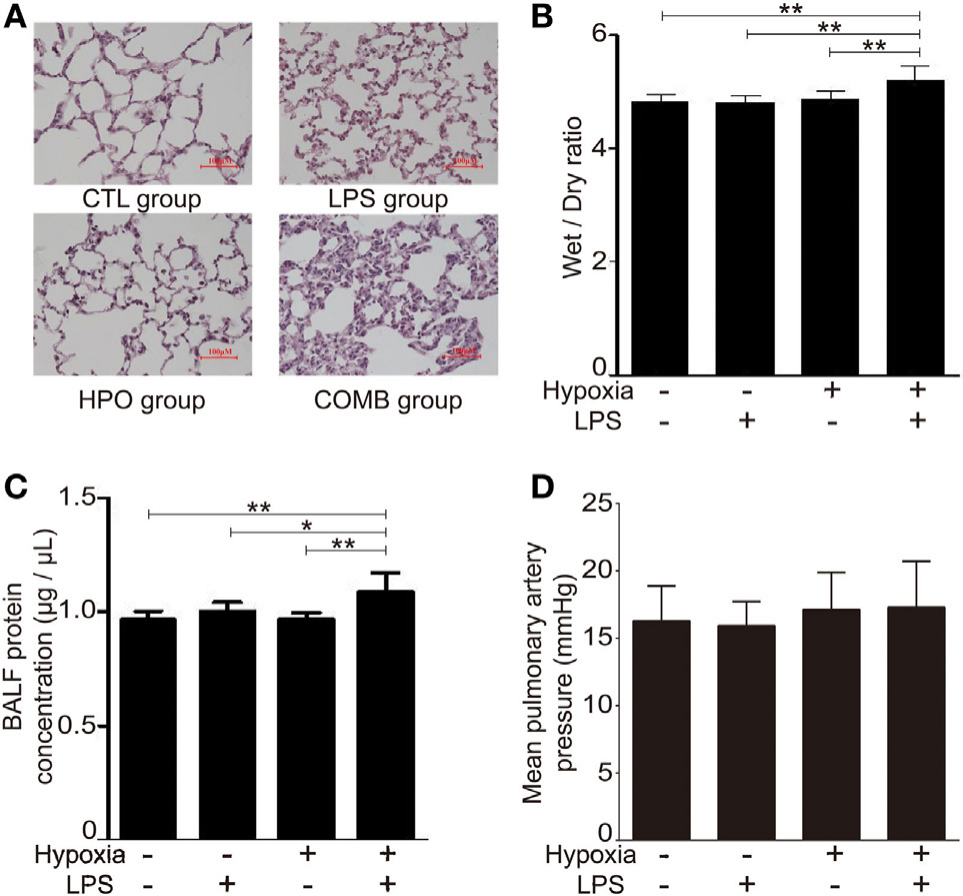
Acute lung injury models were induced by hypoxia combined with lipopolysaccharides (LPS). Histopathologic sections of lung tissues (hematoxylin and eosin staining, ×100) showed that the number of inflammatory cells in the alveolar cavity and the degree of alveolar septum engorgement in CTL (100 μl normal saline) group, LPS (0.5 mg/kg) group, HPO (5,000 m) group, and COMB group **(A)**. The Wet-to-Dry ratio of rat lungs was detected at 6 h after rats were exposed to different stimulation (*n* = 8) **(B)**. The protein concentration in bronchi alveolar lavage fluid (BALF) was determined in triplicates using BCA method (*n* = 10) **(C)**. Mean pulmonary artery pressure (mPAP) values were detected in rats (*n* = 10) **(D)**. Data are presented as mean ± SD. *p < 0.05 and **p < 0.01 between groups.

To explore the interaction and combined effect of sepsis and hypoxia in the pathogenesis of ALI, we established a novel rat ALI model that was induced by systemic LPS with hypoxia exposure (see Materials and Methods). After being subjected to this ALI model, histopathologic evaluation of the lung tissue was performed using H&E staining. H&E data showed the presence of a small number of inflammatory cells in the alveolar cavity and slightly widened alveolar septum in the LPS group and hypoxia group, but with no significant alveolar septum engorgement or disorganization of alveolar structure; in the COMB group, however, the number of inflammatory cells was significantly increased, along with significant alveolar septum engorgement and serious disorganization of alveolar structure (Figure 1A).

We evaluated the lung W/D ratio of ALI rats. Our data showed that the lung W/D ratio of LPS group and hypoxia group were similar to the control group (*n* = 8 per group, *p* > 0.05), whereas W/D significantly increased in the COMB group (*n* = 8, *p* < 0.01) (Figure 1B); this suggests a more severe pulmonary edema occurred in COMB group. Furthermore, the COMB group had significantly higher protein concentration in its BALF compared to the other two treated groups and controls (*n* = 10 per group, *p* < 0.01) (Figure 1C), indicating a more severe protein leakage in the COMB group. However, there was no significant difference of mPAP across the two treated groups and control group (*n* = 10 per group, *p* > 0.05) (Figure 1D).

### Hypoxia Exacerbated Inflammatory Cytokine Response in ALI Rats and Cultured NR8383 Cells

**Figure 2:**
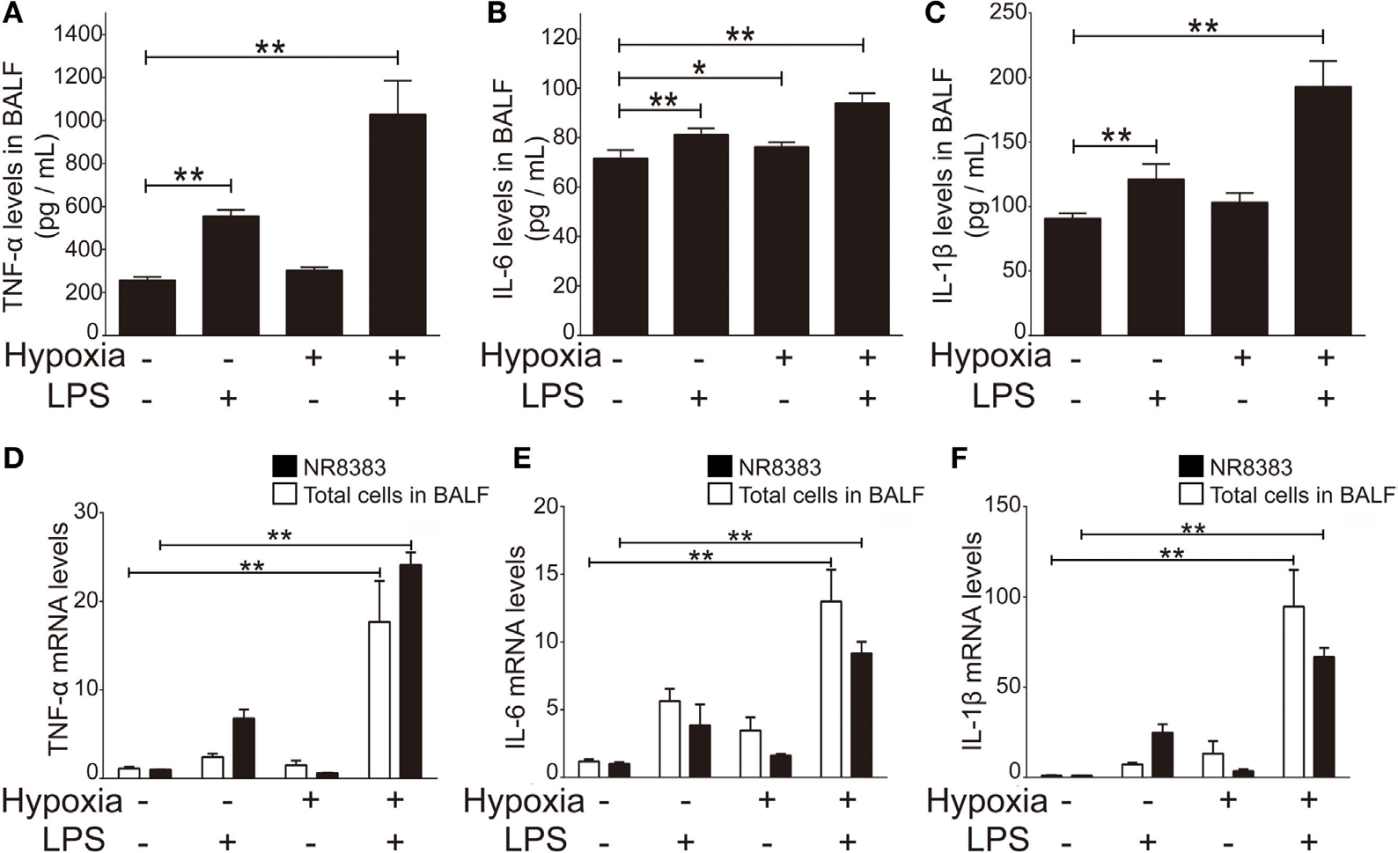
The production of inflammatory cytokines in the bronchi alveolar lavage fluid (BALF) of rats in four groups. The inflammatory cytokines, tumor necrosis factor alpha (TNF-α), interleukin-1 beta (IL-1β), and interleukin-6 (IL-6) in the BALF of rats were detected by enzyme-linked immune sorbent assay (*n* = 10) **(A–C)**. The transcription level changes of TNF-α, IL-6, and IL-1β mRNA was determined by real-time quantitative PCR in NR8383 (*n* = 3) and the total cells in BALF (*n* = 5). The values of controls were normalized to 1 **(D–F)**. Data are presented as mean ± SD. *p < 0.05 and **p < 0.01 between two groups.

To investigate the inflammatory profiles of ALI rats in response to LPS-, hypoxia-, or combined-stimulation, we measured the plasma level of inflammatory cytokines (TNF-α, IL-1β, and IL-6) in BALF of ALI rats. Our data showed the levels of TNF-α, IL-1β, and IL-6 were significantly increased in all ALI groups compared to controls (*p* < 0.01); notably, the levels of inflammatory cytokines in the COMB group were higher than either the LPS group or HPO group (*n* = 5 per group) (Figures 2A–C). Furthermore, to evaluate the transcription level of TNF-α, IL-1β, and IL-6, we measured mRNA levels in total cells of BALF, by RT-qPCR. Quantitative data showed mRNA levels of all three were significantly increased in COMB group compared to the other ALI groups and controls (*n* = 5 per group, *p* < 0.01) (Figures 2D–F). Last, we verified above *in vivo* findings in the *in vitro* setting of cultured macrophage line NR8383. Our data showed that in line with *in vivo* findings, the mRNA levels of TNF-α, IL-1β, and IL-6 were significantly higher in COMB group stimulated NR8383 cells compared to LPS, HPO, and CTL groups in three independent experiments (*p* < 0.01) (Figures 2D–F). Together, our data suggested that COMB stimulation produces a significantly stronger inflammatory response *in vivo* and *in vitro*.

### GO Consortium of the Altered Genes in the BALF Cells of ALI Rats

**Figure 3:**
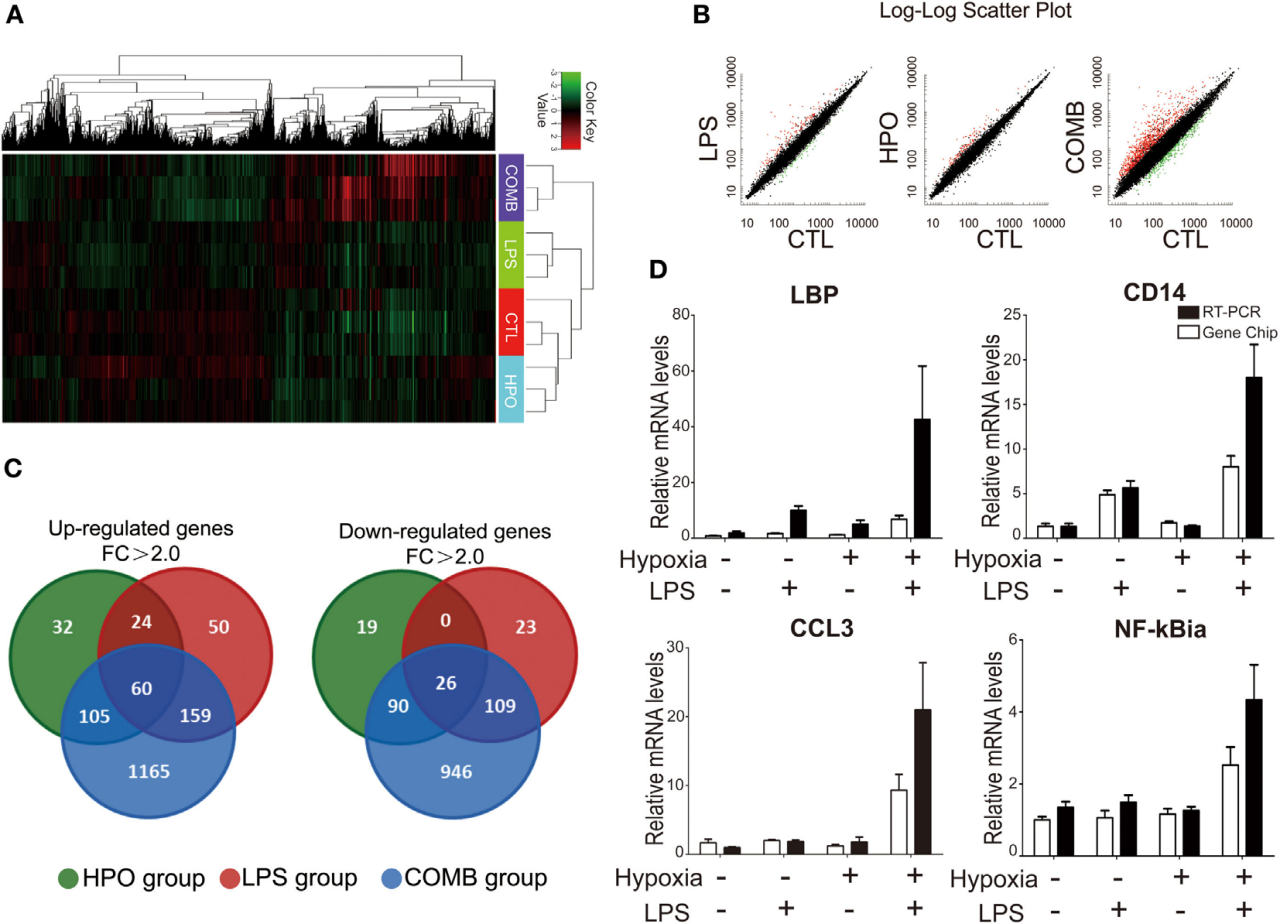
Differential gene expression profiles were identified in the total cells of bronchi alveolar lavage fluid (BALF) by high-throughput microarray analysis. Before the gene chip detection, we mixed three animal samples into one pooled sample and total of three pooled samples were detected in each group. The clustering display was generated by Chip software with two-way data clustering. Each column represents an individual gene, and each row corresponds to an individual array. Gene expression values were standardized and color-coded relative to the mean (green, values less than the mean; red, values greater than the mean), the gene expression profiles of the COMB group are significant different from the other three groups **(A)**. We used the Scatter Plot to show the differential patterns of gene expression in the four groups **(B)**. We used a Venn diagram to show the distribution patterns of gene changes in lipopolysaccharides (LPS) group, HPO group, and COMB group **(C)**. Microarray findings of gene expression pattern were validated by using real-time quantitative PCR (RT-qPCR) **(D)**.

To evaluate the interaction of LPS and hypoxia on the expression of genes participating in ALI development, we used microarray analysis to measure gene expression profiles in the BALF cells of ALI rats. The expression values of each gene were standardized and color-coded relative to the mean (green, values less than the mean; red, values greater than the mean). The microarray data showed that the three ALI groups and controls had significantly distinguished gene expression profiles (Figure 3A). Scatter Plot data further revealed the differential patterns of gene expression in the four groups, and showed that the COMB group had drastically modulated gene expressions compared to the LPS and HPO groups (Figure 3B). Furthermore, we used a Venn diagram to show the distribution patterns of gene changes and the number of the genes regulated and shared among different ALI groups: our data showed a total of 1,595 genes (1,489 in COMB group, 293 in LPS group, and 221 in hypoxia group) were upregulated. Among these genes, 60 (3.8%) genes were upregulated in all three ALI groups. Meanwhile, of a total of 1,213 genes (1,171 in COMB group, 158 in LPS group, and 135 in hypoxia group) that were downregulated, only 26 (2%) of these genes were commonly downregulated in all ALI groups (Figure 3C). Finally, we validated the above microarray findings using RT-qPCR and confirmed the expression patterns of selected genes (i.e., LBP, CD14, CCL3, and NF-kBia), as evidenced by a similar fold change that closely matched the microarray data for the genes tested (Figure 3D). Importantly, the RT-qPCR data suggested that the combined stimulation of LPS and hypoxia had a synergistic effect on gene expression levels in ALI.

**Figure 4:**
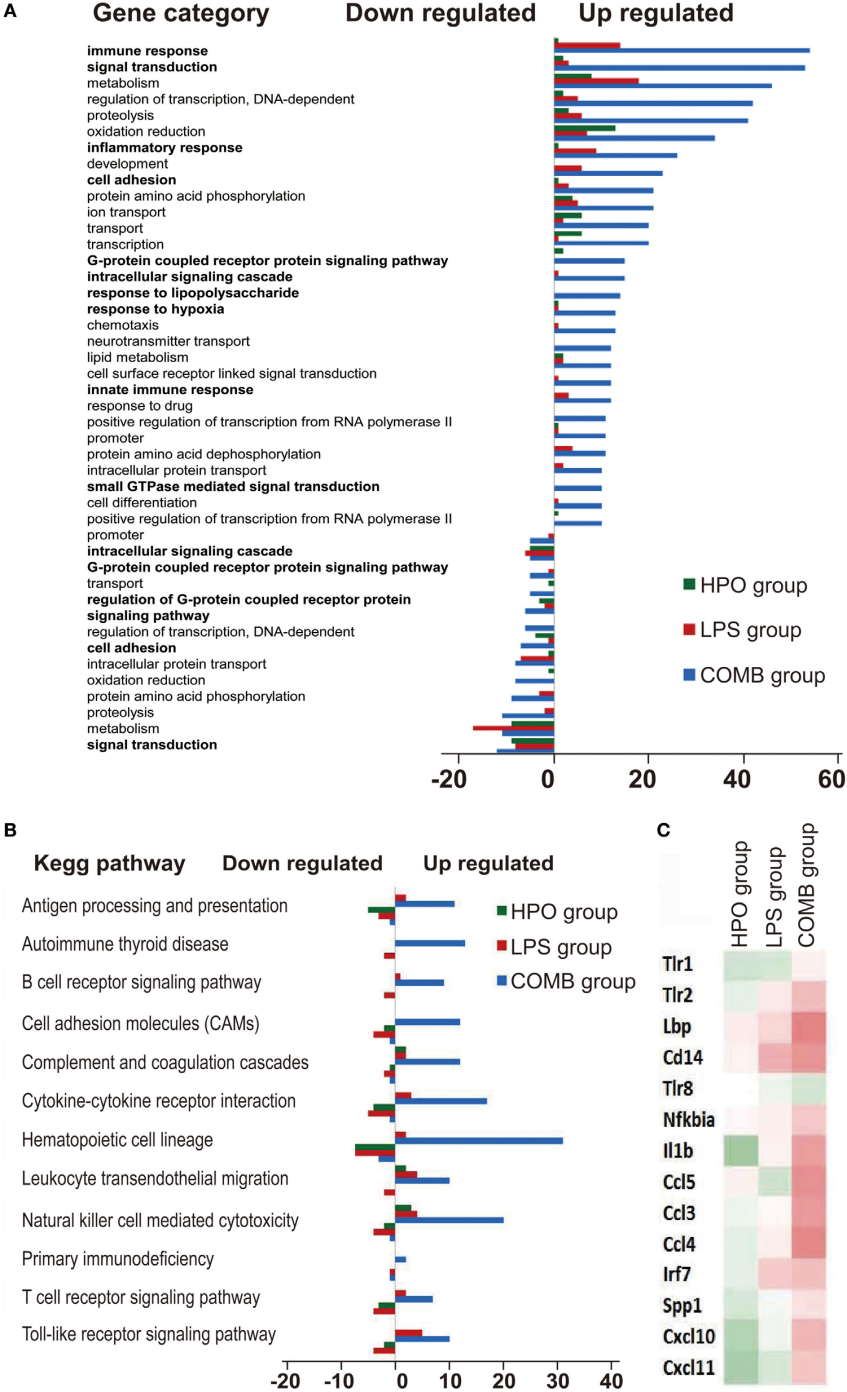
Gene ontology (GO) consortium and pathway analysis of differentially expressed genes. We used GO cluster analysis to classify the differentially expressed genes in the COMB group **(A)**. Meanwhile, we also used KEGG signal pathway classification analysis to find the mainly changed signal pathways **(B)**. The gene expression values in the TLR signal pathways were standardized and color-coded relative to the mean, and each row represents an individual gene (green, values less than the mean; red, values greater than the mean) **(C)**.

To mechanistically understand the interaction of inflammation and hypoxia, we classified the differentially expressed genes in the COMB group ALI into functional categories by three different analysis approaches. First, the GO cluster analysis showed (i) the upregulated 1,489 genes in COMB group belong to functional categories of immune response, signal transduction, inflammatory response, cell adhesion, G-protein coupled receptor protein signaling pathway, intracellular signaling cascade, response to LPS, response to hypoxia, cell adhesion, and so on and (ii) the downregulated 1,171 genes in COMB group related to intracellular signaling cascade, signaling pathway, cell adhesion, signal transduction, etc. (Figure 4A). Second, the KEGG pathway analysis showed those genes are mostly clustered in categories involved in inflammatory responses, such as the TLR signaling pathway, antigen processing and presentation, B cell receptor signaling pathway, and cell adhesion molecules (Figure 4B). Furthermore, to quantify the degree of altered genes in the TLR signaling pathway, we standardized and color-coded the gene expression values relative to their mean values. As shown in Figure 4C, each row represents an individual gene, with green representing values less than the mean and red representing values greater than the mean. Quantitative data showed that the expression of genes in the COMB group of the TLR signaling pathway were significantly higher than in the other two ALI groups.

### Hypoxia Facilitated Expression of Inflammatory Cytokines *via* the TLR4 Signaling Pathway

**Figure 5:**
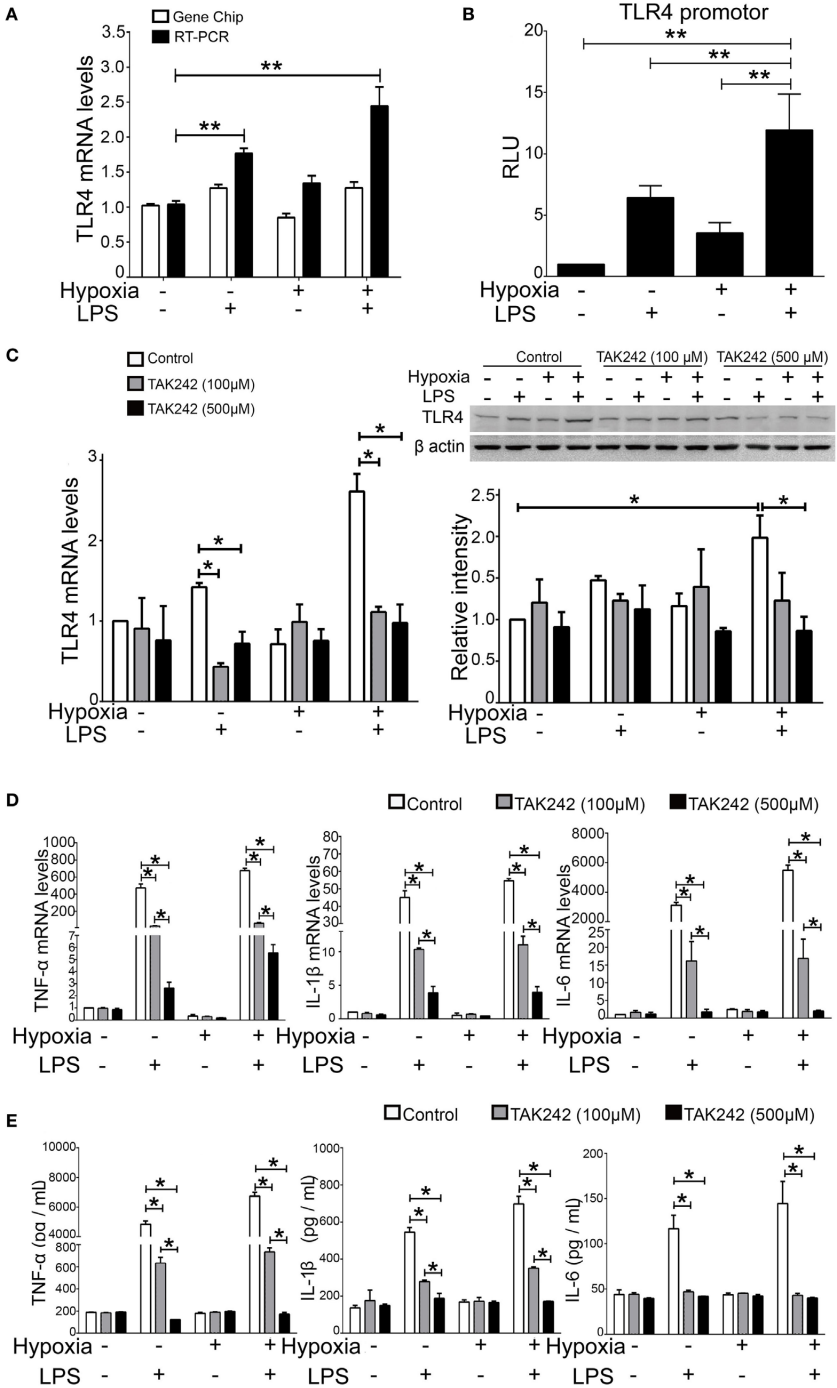
Toll-like receptor 4 (TLR4) signal pathway play an important role in the production of inflammatory cytokines. The microarray result of TLR4 expression pattern was validated by real-time quantitative PCR (RT-qPCR) **(A)**. The promoter activity of the TLR4 gene was detected by reporter assays in NR8383 cells **(B)**. The inhibitory effect of TAK-242 on the TLR4 mRNA and protein expression was detected by RT-qPCR and Western Blot in NR8383 cells **(C)**. The transcription levels of inflammatory cytokines, such as tumor necrosis factor alpha (TNF-α), interleukin-1 beta (IL-1β), and interleukin-6 (IL-6), were detected by RT-qPCR in NR8383 cells **(D)**; and their production levels in the culture medium were detected by enzyme-linked immune sorbent assay **(E)**. Data are presented as mean ± SD of three independent experimental repeats. *p < 0.05 and **p < 0.01 between groups.

Our GO Consortium and Pathway Analysis data suggest that the TLR4 signaling pathway drastically changed across all ALI groups. To further validate this finding, we tested the regulatory role of TLR4 in response to stimulation by LPS and/or hypoxia in NR8383 cells, an *in vitro* system. First, the RT-qPCR assay showed that the TLR4 mRNA level was significantly increased in all ALI groups of rats, which is in line with the microarray results (Figure 5A). Second, to evaluate the role and possible crosstalk of hypoxia and LPS in the regulation of transactivation of TLR4, we evaluated the promoter activities of TLR4 after different stimulations in reporter assays. The results showed that the promoter activities of the TLR4 gene were significantly increased in all groups. Importantly, the promoter activity of TLR4 in the COMB group was higher than LPS or hypoxia alone, showing a synergistic effect (Figure 5B). Third, to clarify the role of the TLR4 signaling pathway in the pathogenesis of ALI, we examined the inhibitory effect of TAK-242 on the production of an inflammatory cytokine *in vitro* as previously described (40). The cultured NR8383 cells were pretreated with TAK-242 (100 or 500 µM) for 24 h (41), then washed with PBS. The cells in LPS group were then stimulated with LPS (0.5 µg/ml), whereas cells in the HPO group were stimulated with 5% O_2_; cells in COMB group suffered both stimuli. TAK-242 significantly inhibited TLR4 mRNA and protein expression in the LPS and COMB group (Figure 5C). TAK-242 also inhibited the expression of inflammatory cytokines, TNF-α, IL-1β, and IL-6 (Figure 5E), as well as their transcription levels (Figure 5D). The *in vitro* results indicate to us that TLR4 blockade by TAK-242 could effectively suppress inflammatory responses induced by LPS or hypoxia combined LPS, but the impact of hypoxia alone is not obvious. Importantly, the above findings, together with our GO Consortium and Pathway Analysis data, suggest that the macrophageal TLR4 signaling pathway plays an important role in alveolar inflammation as a link between hypoxia and inflammation.

### HIF-1α Regulated Inflammatory Response of Cytokines *via* TLR4 Activity

**Figure 6:**
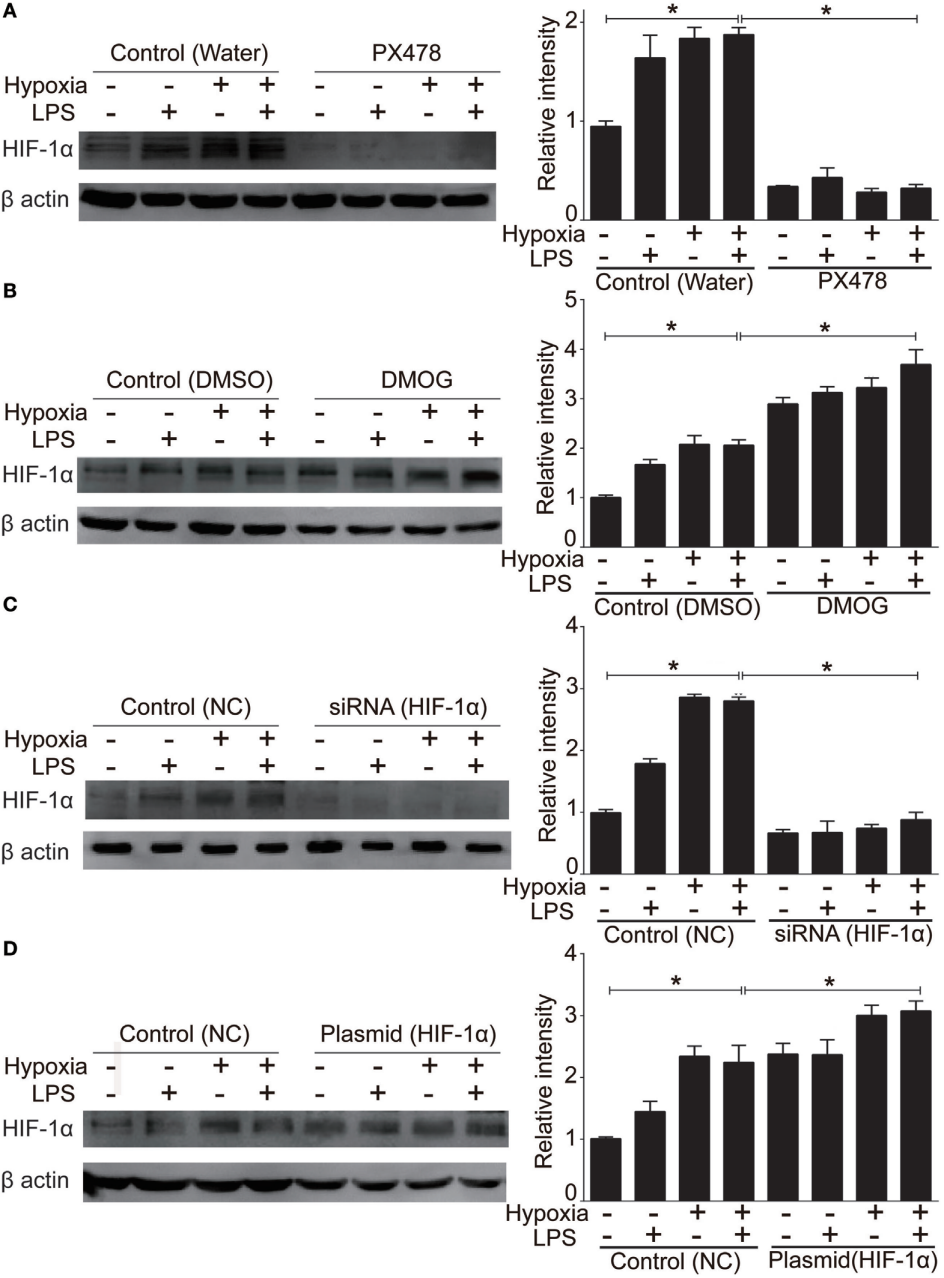
Manipulation of the protein level of hypoxia-inducible factor 1 alpha (HIF-1α) in NR8383. We used 50 µM PX478 treatment for 20 h **(A)** or transfection of a small interfering RNA (siRNA) specific for HIF-1α for 48 h **(C)** to downregulate the HIF-1α protein level in NR8383 cells. We used 1 mM DMOG treatment for 8 h **(B)** or transfection of a plasmid specific for HIF-1α for 48 h **(D)** to upregulate the HIF-1α protein level in NR8383 cells. Data are presented as mean ± SD of three independent experimental repeats. *p < 0.05 between groups.

**Figure 7:**
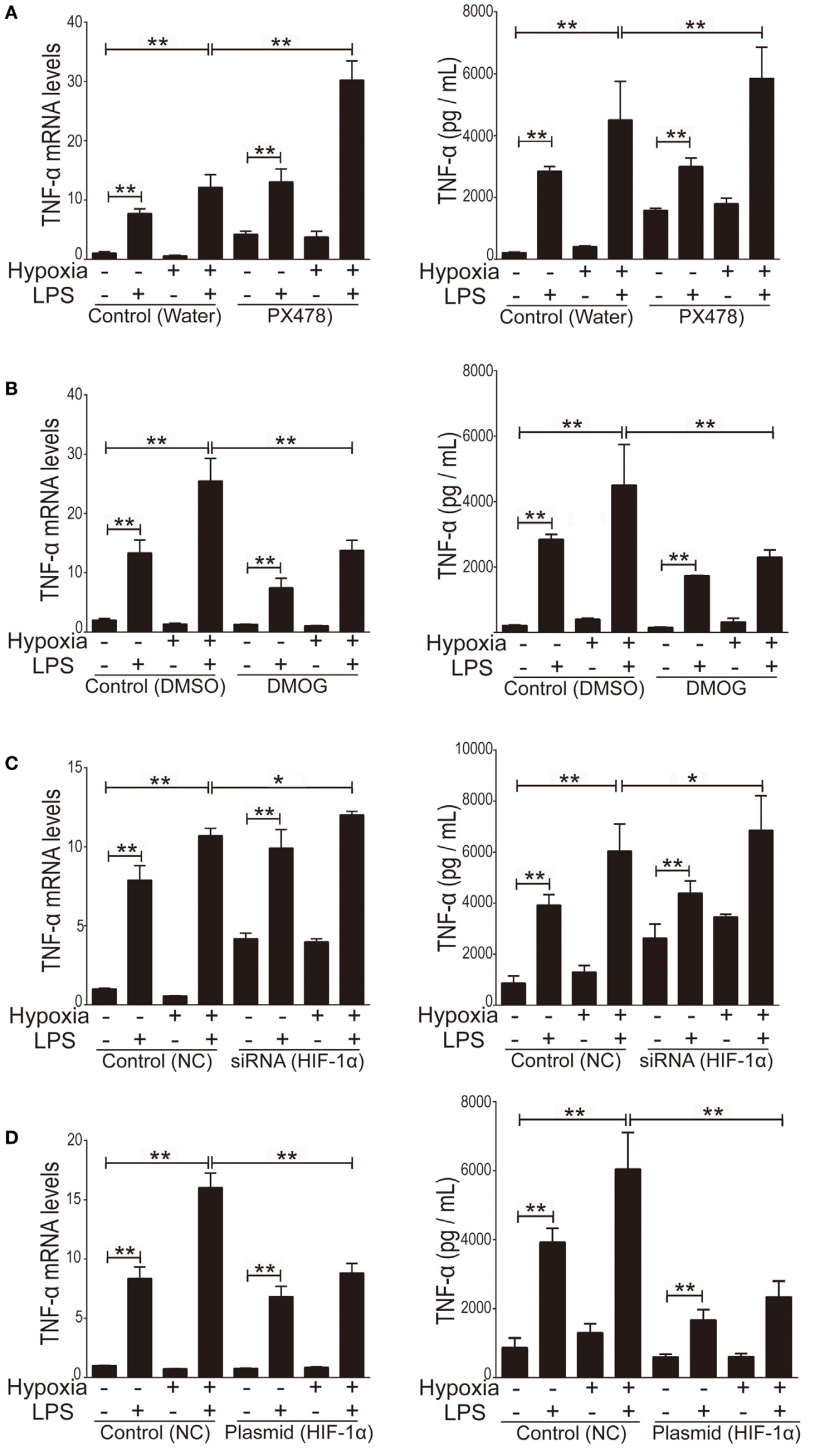
The effect of manipulation of hypoxia-inducible factor 1 alpha (HIF-1α) on the production of inflammatory cytokine tumor necrosis factor alpha (TNF-α). We used 50 µM PX478 treatment for 20 h **(A)** or transfection of a small interfering RNA (siRNA) specific for HIF-1α for 48 h **(C)** to downregulate the HIF-1α protein level in NR8383 cells. We used 1 mM DMOG treatment for 8 h **(B)** or transfection of a plasmid specific for HIF-1α for 48 h **(D)** to upregulate the HIF-1α protein level in NR8383 cells. Then we detected TNF-α levels in the culture media by using enzyme-linked immune sorbent assay and its mRNA expression in NR8383 cells by using real-time quantitative PCR. Data are presented as mean ± SD of three independent experimental repeats. **p < 0.01 between groups.

**Figure 8:**
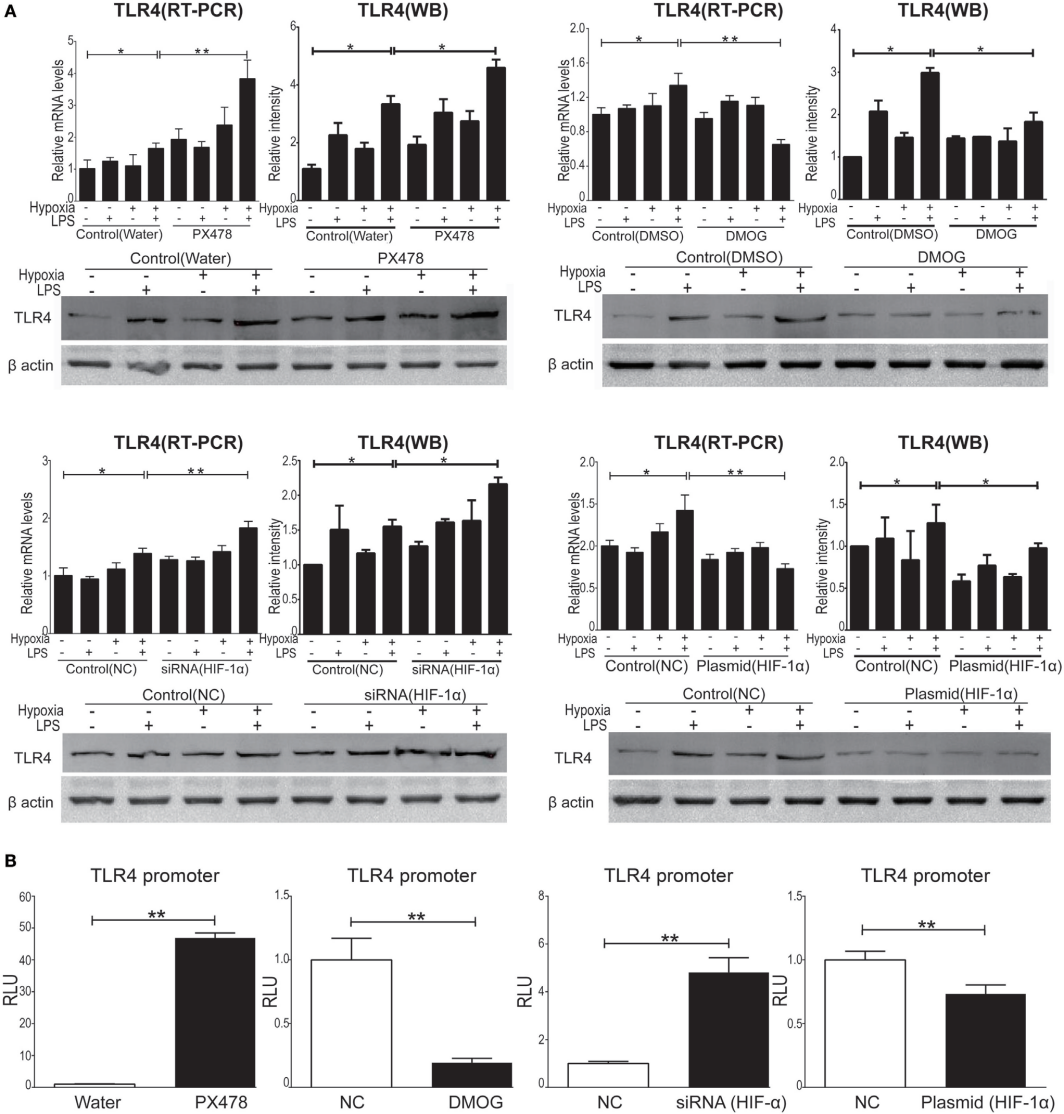
Hypoxia-inducible factor 1 alpha (HIF-1α) manipulation regulated the expression of toll-like receptor 4 (TLR4) in NR8383. We manipulated HIF-1α by using PX478 or small interfering RNA targeting HIF-1α transfection to downregulated HIF-1α protein level and by using DMOG or a plasmid specific for HIF-1α transfection to upregulate HIF-1α protein level in NR8383 cells. TLR4 mRNA and protein levels were detected by qPCR and Western Blot **(A)**. TLR4 promoter activity was detected by reporter assay **(B)**. Data are presented as mean ± SD of three independent experimental repeats. *p < 0.05 and **p < 0.01 between groups.

To evaluate HIF-1α as a therapeutic target of ALI and to test the treatment effects of HIF-1α augmentations against both inflammation and hypoxia in ALI, we used four HIF-1α related manipulatory approaches to treat cultured NR8383 cells: (i) the HIF-1α inhibitor PX-478; (ii) a HIF-1α-specific siRNA; (iii) the HIF-1α agonist DMOG; and (iv) a HIF-1α overexpression plasmid. Expression and transcription levels of HIF-1α and the inflammatory cytokine TNF-α were measured after manipulation of HIF-1α. First, it was shown that in cultured NR8383 cells treated with 50 µM PX478 for 20 h (38), the protein levels of HIF-1α were significantly decreased compared to controls (Figure 6A). Similarly, treatment of HIF-1α-specific siRNA for 48 h significantly reduced the protein levels of HIF-1α (Figure 6C). Furthermore, treatment of 1 mM DMOG for 8 h significantly increased the protein level of HIF-1α in NR8383 cells (42) (Figure 6B). Similarly, treatment of HIF-1α-overexpression plasmid for 48 h significantly increased the protein levels of HIF-1α (Figure 6D). RT-qPCR and ELISA were used to detect the mRNA or protein levels of TNF-α in the cells and culture media, correspondingly. The production of inflammatory cytokine (TNF-α) was significantly increased after we inhibited the expression of HIF-1α (Figures 7A,C). On the contrary, increasing the levels of HIF-1α significantly decreased the protein and mRNA levels of TNF-α (Figures 7B,D). To further study the mechanisms of the above HIF-1α effects on cytokine expression and the potential role of TLR4, we then measured the promoter activities of the TLR4 gene using the same aforementioned treatments and paradigms. The reporter assay showed that HIF-1α inhibition increased the promoter activities of the TLR4 gene (Figure 8B), which accompanied a subsequent upregulation of TLR4 mRNA and protein expression (Figure 8A). By contrast, augmentation of HIF-1α suppressed the promoter activities of the TLR4 gene (Figure 8B) and downregulated both TLR4 gene and protein expression (Figure 8A). Together, our data indicate that targeting HIF-1α can effectively manipulate the expression of inflammatory cytokines, *via* altering the promoter activities of the TLR4 gene.

### Preconditioning of HIF-1α Augmentation Decreased Inflammation

**Figure 9:**
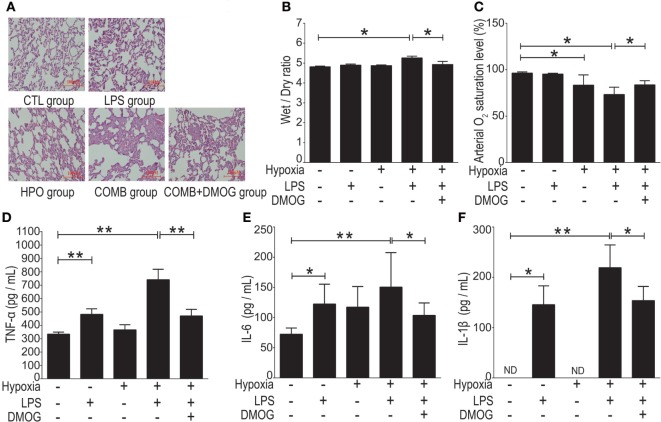
Preconditioning of hypoxia-inducible factor 1 alpha (HIF-1α) alleviated acute lung injury (ALI) induced by combined lipopolysaccharides (LPS)/hypoxia stimuli in rats. We gave an i.p. injection DMOG (50 mg/kg, body weight) to rats 8 h before subjected to the ALI model of COMB stimulation. Hematoxylin and eosin (H&E) staining showed the number of inflammatory cells in the alveolar cavity and the degree of the alveolar septum engorgement (H&E, ×100) **(A)**. The Wet-to-Dry ratio of rat lungs was detected 6 h after rats’ exposure to different stimulation in the four groups (*n* = 10) **(B)**. The arterial oxygen saturation of the rats was detected by blood gas analyzer (*n* = 10) **(C)**. The inflammatory cytokine levels of tumor necrosis factor alpha (TNF-α), interleukin-1 beta (IL-1β), and interleukin-6 (IL-6) in the plasma of rats were detected by enzyme-linked immune sorbent assay (*n* = 10) **(D–F)**. Data are presented as mean ± SD. *p < 0.05 and **p < 0.01 between two groups.

To evaluate the *in vivo* effect of HIF-1α in ALI, we augmented HIF-1α with pretreatment with DMOG (50 mg/kg, single i.p.) in rats 8 h before being subjected to the ALI model COMB stimulation; subsequently, pathological evaluation and cytokine expression were evaluated. Histological H&E staining showed that the number of inflammatory cells in the alveolar cavity and level of septum engorgement of DMOG-pretreated ALI rats were significantly lower than those with only vehicle pretreatment (Figure 9A). Also, the W/D ratio in lung tissue of the DMOG pretreated group was significantly decreased compared to the vehicle pretreated ALI group (*n* = 10 per group, *p* < 0.05) (Figure 9B). Furthermore, the arterial oxygen saturation of the DMOG pretreated group was significantly higher than the vehicle pretreated ALI group (*n* = 10 per group, *p* < 0.05) (Figure 9C). Finally, the plasma levels of inflammatory cytokines TNF-α, IL-1β, and IL-6 were significantly lower than those in the vehicle-pretreated group (*n* = 10 per group) (Figures 9D–F). Together, we see that the preconditioning of HIF-1α by augmentation with DMOG decreased inflammatory impairment in ALI rats.

## Discussion

Sepsis is one of the most common causes of ALI, with LPS being the most important biological mediator as they induce the secretion of inflammatory cytokines, such as TNF-α, IL-1β, and IL-6 in response to bacterial toxins. In clinic settings, hypoxia commonly coexists with sepsis, therefore, the understanding of the interaction between hypoxia and inflammation in ALI is of great importance for the treatment of ALI. To evaluate their possible crosstalk and its effect on ALI, we established a new ALI rat model and tested the *in vivo* and *in vitro* modulatory effects of hypoxia on inflammatory responses. Here, we demonstrated: (i) LPS upregulated inflammatory genes in the BALF cells could be further aggravated by hypoxia; (ii) hypoxia-induced exacerbation of alveolar macrophageal inflammation was mediated by TLR4; conversely, this could be suppressed by TLR4 antagonist TAK-242, suggesting TLR4 as a central link between inflammation and hypoxia in ALI; (iii) manipulation of HIF-1α altered the development of ALI, *via* suppressing the promoter activity of *TLR4 gene*; and importantly, and (iv) the preconditioning augmentation of HIF-1α by HIF hydroxylase inhibitor DMOG protected against both inflammatory and hypoxic processes in ALI.

### Hypoxia Affects the Development of ALI

The effect of hypoxia on ALI is unclear with debatable findings from limited studies. For instance, Agorreta et al. used LPS (0.1 mg/kg, single i.p.) combined with normobaric hypoxia (9% O_2_) exposure in rats to mimick the pathophysiological changes of acute phase of ALI (43). However, in their study, hypoxia exposure of rats was conducted by placing animals in an experimental chamber (with low oxygen gas flow), which would inevitably raise a concern that rats might re-oxygenated upon sacrifice and sampling after finishing their hypoxia exposure. Indeed, hypoxia-re-oxygenation *per se* can cause reactive oxygen spices production and contribute to additional lung injury (44, 45). Therefore, we conducted the entire rat ALI experiments as well as sample collections in a large-scale hypobaric hypoxia chamber (46), which mimics oxygen levels at a high altitude of 5,000 m (about 11% O_2_) to avoid hypoxia-re-oxygenation effects. Taking advantage of a large-scale hypobaric hypoxia chamber, we confirmed the findings from Agorreta et al. (43) and further concluded that even a low-dose (0.5 mg/kg) LPS (versus a conventional dose range of 5–45 mg/kg) was sufficient to cause ALI in rats when combined with hypoxic exposure (Figure 1), whereas such a low-dose LPS or hypoxia alone was not. The gene expression profiles of BALF cells showed that hypoxia exacerbated LPS-inducible inflammatory changes (Figures 3 and 4). Our findings for the first time reveal that the synergistic effects of hypoxia with inflammation on ALI mostly affect inflammatory and immune responses and contribute to impairment of the alveolar-capillary membrane integrity in ALI. With this in mind, the oxygenation and ventilation treatment need to be emphasized in clinic settings, like acute respiratory distress syndrome (ARDS) that is characterized by acute bilateral inflammatory pulmonary infiltrates and severe hypoxia (47).

### HAPE and ALI

Though it is out of the scope of this study, the effect of hypoxia on ALI is also of importance regarding the role of HAPE in the etiology of ALI. According to the classical pathophysiological theory of HAPE, acute hypoxia causes contraction of pulmonary vessels that results in a redistribution of pulmonary blood pouring into less constricted vessels (namely, this leads to a “stress failure”) and physically breakdown of the alveolar-capillary barrier (48, 49). In the latest revised criteria of ARDS, left atrial hypertension causing hydrostatic pulmonary edema was removed, due to the distinction that inflammatory lung disease and elevated left atrial pressures are not mutually exclusive (50). Of note, in the present study, we did not observe significant mPAP change (Figure 1D) in all three ALI rat groups compared to controls, though they had significant pulmonary inflammatory infiltration (Figure 1) and cytokine changes (Figure 2). The hemodynamic findings from our combined hypoxia/LPS model of ALI suggest that inflammation—rather than pulmonary hypertension—is crucial as a central link of ALI pathophysiology. In addition, our findings support the inflammatory mechanism for the development of HAPE. Indeed, it has rarely been successful to establish a stable rodent model of HAPE by hypoxia exposure *per se*. Since risk factors including inflammation and hypoxia are found in most HAPE patients (15, 16), the synergistic effect of inflammation and hypoxia exposure may be critical in the development of HAPE (51–53). People with inflammatory conditions, for example, upper respiratory tract infection, should be advised to avoid high altitude exposure regarding the concern of HAPE. The potential protective HIF-1 manipulation (for details, see the following discussion) may be introduced for HAPE intervention.

### The TLR4 Pathway in ALI

Among the genes and related biological pathways that we tested, no such drastic changes in the TLR signaling pathway was found in the ALI rats induced by either LPS (Table 2) or hypoxia (Table 3) alone as it was induced by combined LPS and hypoxia (Table 4), suggesting that hypoxia and LPS have synergistic effects on the TLR signaling pathway and that TLR4 plays a crucial role in bridging the interaction between hypoxia and inflammation. TLR4 is a transmembrane protein which belongs to the pattern recognition receptor family and acts as the key node in the LPS-inducible inflammatory responses (54). Our microarray analysis data also indicated that hypoxia exacerbated LPS-induced inflammatory responses, particularly upregulating expression of genes downstream of TLR4 pathways. Previous reports showed that LPS *per se* can induce the expression of TLR4 in both animal and cellular studies (55–57). Meanwhile, the regulatory roles of hypoxia on TLR4 were found from studies on human trophoblast cells (26), endothelial cells (58), kidneys (59), as well as respiratory epithelial cells (60). In this study, we demonstrated that TLR4 mRNA increased in rat BALF cells after 6 h hypoxia exposure (Figure 5), as the first *in vivo* data presented regarding the effects of hypoxia on the regulation of TLR4 mRNA in BALF cells of ALI animals. There were discrepant results reported regarding the role of hypoxic regulation on TLR4 expression (26, 58–61), which may stem from experimental differences, such as the difference in the duration of hypoxic exposure, hypoxic severity, or cell type.

**Table 2:** All of the 29 differentially expressed pathways in bronchi alveolar lavage fluid cells in the lipopolysaccharides group.

Pathway	Input number	*p*-Value	*q*-Value
Antigen processing and presentation	9	2.74E−07	1.81E−05
Cytokine–cytokine receptor interaction	32	3.18E−06	1.05E−04
MAPK signaling pathway	16	5.53E−06	1.22E−04
Nicotinate and nicotinamide metabolism	5	1.35E−05	2.23E−04
Glutathione metabolism	8	1.82E−04	0.002
Caffeine metabolism	1	2.38E−04	0.003
Pantothenate and CoA biosynthesis	3	3.62E−04	0.003
Metabolism of xenobiotics by cytochrome P450	6	4.27E−04	0.004
Graft-versus-host disease	8	4.98E−04	0.004
Cell adhesion molecules	19	0.001	0.004
Drug metabolism—cytochrome P450	8	0.001	0.004
Type I diabetes mellitus	9	0.001	0.004
Hematopoietic cell lineage	18	0.001	0.005
Biosynthesis of unsaturated fatty acids	1	0.002	0.010
Apoptosis	8	0.002	0.010
Leukocyte transendothelial migration	12	0.003	0.014
ABC transporters—general	3	0.004	0.014
Jak–STAT signaling pathway	9	0.006	0.020
Natural killer cell mediated cytotoxicity	11	0.006	0.020
Valine, leucine and isoleucine degradation	3	0.006	0.020
Drug metabolism—other enzymes	2	0.007	0.022
Arachidonic acid metabolism	8	0.010	0.031
Allograft rejection	6	0.011	0.032
Retinol metabolism	3	0.013	0.034
Calcium signaling pathway	12	0.014	0.034
Autoimmune thyroid disease	5	0.014	0.034
Long-term depression	6	0.014	0.034
VEGF signaling pathway	4	0.015	0.035
Riboflavin metabolism	2	0.021	0.047

**Table 3:** All of the 17 differentially expressed pathways in bronchi alveolar lavage fluid cells in the HPO group.

Pathway	Input number	*p*-Value	*q*-Value
Drug metabolism—cytochrome P450	8	5.38E−14	2.64E−12
Metabolism of xenobiotics by cytochrome P450	6	1.59E−12	3.89E−11
Arachidonic acid metabolism	8	1.50E−06	2.45E−05
Retinol metabolism	3	2.22E−06	2.72E−05
Alkaloid biosynthesis II	3	4.07E−05	3.99E−04
Complement and coagulation cascades	11	1.24E−04	1.01E−03
Histidine metabolism	3	4.41E−04	3.09E−03
Glutathione metabolism	8	0.002	0.01
Drug metabolism—other enzymes	2	0.002	0.011
MAPK signaling pathway	16	0.006	0.029
Glycolysis/gluconeogenesis	3	0.008	0.038
3-Chloroacrylic acid degradation	1	0.011	0.042
Riboflavin metabolism	2	0.011	0.042
Tight junction	9	0.014	0.044
Sulfur metabolism	1	0.014	0.044
Limonene and pinene degradation	1	0.015	0.044
Pantothenate and CoA biosynthesis	3	0.015	0.044

**Table 4:** Top 30 of 118 differentially expressed pathways in bronchi alveolar lavage fluid cells in the COMB group.

Pathway	Input number	*p*-Value	*q*-Value
Cytokine-cytokine receptor interaction	32	6.54E−23	8.77E−22
Hematopoietic cell lineage	18	4.76E−18	3.99E−17
Cell adhesion molecules	19	1.10E−13	7.39E−13
Complement and coagulation cascades	11	1.20E−09	4.75E−09
Toll-like receptor signaling pathway	11	3.58E−08	9.23E−08
Leukocyte transendothelial migration	12	3.94E−08	9.77E−08
Glutathione metabolism	8	5.97E−08	1.38E−07
Type I diabetes mellitus	9	2.37E−07	4.97E−07
MAPK signaling pathway	16	3.52E−07	6.94E−07
Adherens junction	9	3.92E−07	7.50E−07
Regulation of actin cytoskeleton	14	5.47E−07	9.40E−07
Arachidonic acid metabolism	8	9.25E−07	1.39E−06
Graft-versus-host disease	8	9.25E−07	1.39E−06
Drug metabolism—cytochrome P450	8	2.49E−06	3.40E−06
Nicotinate and nicotinamide metabolism	5	2.65E−06	3.49E−06
Natural killer cell-mediated cytotoxicity	11	2.66E−06	3.49E−06
Prostate cancer	9	3.81E−06	4.72E−06
Antigen processing and presentation	9	6.90E−06	8.26E−06
Calcium signaling pathway	12	7.84E−06	8.91E−06
Ether lipid metabolism	5	1.87E−05	1.96E−05
Melanogenesis	8	3.64E−05	3.54E−05
Focal adhesion	11	3.78E−05	3.62E−05
Apoptosis	8	4.23E−05	3.88E−05
Tight junction	9	4.36E−05	3.95E−05
Cyanoamino acid metabolism	3	7.26E−05	6.28E−05
Alkaloid biosynthesis II	3	7.26E−05	6.28E−05
Primary immunodeficiency	5	8.39E−05	7.03E−05
Metabolism of xenobiotics by cytochrome P450	6	8.56E−05	7.08E−05
Jak–STAT signaling pathway	9	9.29E−05	7.41E−05

Our *in vivo* finding from BALF cells—namely that more than 80% of immune cells are macrophages—were in accordance with an *in vitro* study that exposed cultured macrophages to hypoxia for 2–8 h (61). There was a very limited study regarding the effect of LPS combined with hypoxia on TLR4. Most recently, a report showed that the pretreatment of hypoxia for 24 h could suppress consequential LPS-induced upregulation of TLR4 mRNA (62). In the present study, we for the first time investigated the effect of simultaneous stimulations of LPS and hypoxia and showed that hypoxia synergistically facilitated LPS-induced TLR4 mRNA expression in rat BALF cells (Figure 5). This synergistic effect of hypoxia is absolutely in opposition to the phenomenon observed in a hypoxia preconditioning study (62), suggesting different mechanisms may be involved in different experimental settings. Alveolar macrophages are the primary cell type in regards to the pulmonary immune and inflammatory responses to LPS and combined LPS/hypoxia stimuli. TLR4 is a vital membrane receptor to trigger LPS signaling, and its expression level correlates with the threshold and susceptibility of LPS response (63). Here, we demonstrated that hypoxia exposure exacerbated LPS response *via* the induced activity of the TLR4 promoter in NR8383 cells. Furthermore, the combined LPS/hypoxia stimuli facilitated the expression of downstream pro-inflammatory cytokines in the TLR singling pathway. Importantly, the selective inhibition of TLR4 pathway by its inhibitor TAK-242 (62) suppressed LPS-induced induction of TLR4 mRNA and protein expression and disrupted the interactions between LPS and TLR4 (40). TAK-242 eliminated the LPS- and combined LPS/hypoxia-induced expression of inflammatory cytokines in a dose dependent manner (Figure 5). The findings support the notion that TLR4 signaling pathway may be a central link between hypoxia and LPS-inducible inflammation in alveolar macrophages.

### Potential Therapeutic Targets of TLR4 and HIF-1α

As TLR4 serves as the central link bridging the effects of inflammation and hypoxia, it could be a potential therapeutic target for ALI. However, clinical results from the application of TLR4 inhibitors, including TAK-242 and eritoran tetrasodium (E5564), failed to show either the inhibition of inflammatory responses or a decrease in the mortality rate in sepsis patients (64, 65). Therefore, alternative therapeutic targets/reagents are in urgent need. HIF-1α is the key transcription factor induced by hypoxic condition (66), which activates target genes involved a broad range of physiology and pathophysiology functions in mammalian cells. Studies suggest a protective role of HIF-1α against pulmonary injuries, however, the effects of HIF-1α modulation on TLR4 expression were discrepancies (67–69). For example, Hu et al. reported a pro-inflammatory effect of HIF-1α in rheumatoid arthritis by provoking TLR signaling (70), whereas other studies indicated that HIF-1α elicited anti-inflammation by promoting FoxP3 expression in T-cells (71) and upregulation of IL-1R-associated kinase-M in monocytes (30). Our findings demonstrate that HIF-1α manipulation suppressed (i) LPS/hypoxia-induced *in vivo* expression of TLR4 *via* inhibition on TLR4 promoter activity (Figure 8) and (ii) *in vitro* macrophageal expression of cytokines induced by LPS, hypoxia, or combined LPS/hypoxia (Figures 6 and 7). Together, our findings show that manipulation of HIF-1α suppressed the LPS/hypoxia-induced TLR4 mRNA and inflammatory cytokines, alleviated lung injury, decreased W/D weight ratio, and increased arterial oxygen saturation after LPS/hypoxia exposure (Figure 9), indicating an anti-inflammatory role of HIF-1α in alveolar macrophage cells *via* TLR4 signal pathway. These findings provide evidence that HIF-1α activators are a promising approach to treat ALI clinically.

## Conclusion

**Figure 10:**
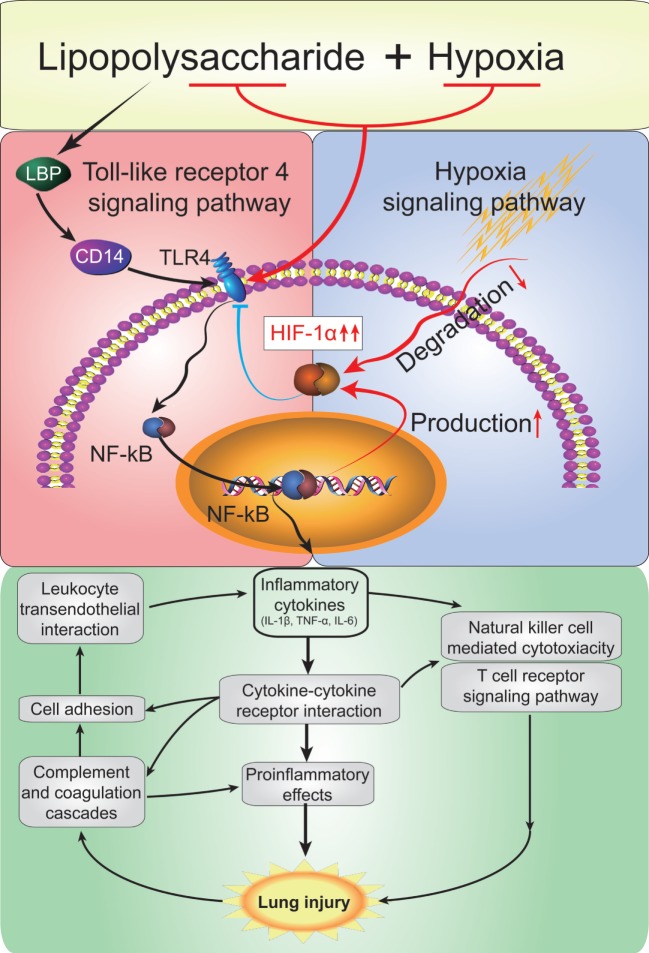
A model depicting the mode that hypoxia exacerbats acute lung injury (ALI) *via* the toll-like receptor 4 (TLR4) signaling pathway and the protective effect of hypoxia-inducible factor 1 alpha (HIF-1α). Acute hypoxia causes a drastic exacerbation of inflammation predisposing ALI. Hypxia potentiates lipopolysaccharides (LPS)-induced cytokine production *via* the TLR4 signaling pathway in alveolar macrophage cells. Moreover, LPS and combined hypoxia stimuli induce compensatory HIF-1α accumulation, in turn, to protect the lungs by supperssing TLR4 expression and attenuating macrophageal inflammatory responses.

Sepsis and hypoxia are both common in clinical settings and the interaction between them has not been fully clarified in ALI. In this study, we have successfully developed a robust rat model of ALI induced by a combined low-dose LPS with acute hypobaric hypoxia. We demonstrate that hypoxia can cause a drastic exacerbation of inflammation predisposing ALI; this is of high clinical relevance when sepsis coexists with hypoxia in patients. Hypoxia potentiates LPS-induced cytokine production *via* the TLR4 signaling pathway in alveolar macrophage cells to impair the alveolar-capillary barrier. Moreover, LPS combined with hypoxia induces compensatory HIF-1α accumulation, in turn, to protect the lungs during ALI by suppressing TLR4 expression and attenuating macrophageal inflammatory responses (Figure 10). Taken together, we show that hypoxia interacts with systemic inflammation to play a crucial role in the development of ALI; thus, the HIF-1α/TLR4 crosstalk pathways emerge as a potential therapeutic target for the treatment of ALI.

## Data Accession

The data has been deposited to Gene Expression Omnibus under accession GSE111241 (https://www.ncbi.nlm.nih.gov/geo/query/acc.cgi?acc=GSE111241) by the secure token ynqfksyyfravxel. These data will be released once this manuscript is accepted.

## Ethics Statement

All study procedures involving animals were performed in accordance with the ethical standards of the Institutional Research Committee, and all animal experiments were approved by the intramural Committee on Ethics Conduct of Animal Studies of the Army Medical University in Chongqing, China.

## Author Contributions

GW contributed to the design of the research, the literature search, the writing of the article, and performed the final revision of the article and its results. GX performed the rat experiments, and analyzed the data. D-WC designed the research, performed the experiments, and analyzed the data. W-XG contributed to the design of the research, analyzed the data, and wrote the article. J-QX contributed to the design of the experiment and wrote the article. H-YS contributed to the design the experiment and the writing of the article. Y-QG helped design the research and the writing of the article.

## Conflict of Interest Statement

The authors declare that the research was conducted in the absence of any commercial or financial relationships that could be construed as a potential conflict of interest.
